# Aminotransferases are associated with insulin resistance and atherosclerosis in rheumatoid arthritis

**DOI:** 10.1186/1471-2261-7-31

**Published:** 2007-10-29

**Authors:** Patrick H Dessein, Angela J Woodiwiss, Barry I Joffe, Gavin R Norton

**Affiliations:** 1 Cardiovascular Pathophysiology and Genomics Research Unit, School of Physiology, Faculty of Health Sciences, University of the Witwatersrand, Johannesburg, South Africa; 2 Rheumatology Unit, Milpark Hospital, Johannesburg, South Africa; 3 Centre for Diabetes and Endocrinology, University of the Witwatersrand, Johannesburg, South Africa

## Abstract

**Background:**

Serum aminotransferase concentrations are reportedly strongly associated with insulin resistance, an established cardiovascular risk factor that is not routinely assessed in clinical practice. We therefore explored the possibility that serum aminotransferase concentrations are as closely related to large artery disease as insulin resistance in rheumatoid arthritis (RA).

**Methods:**

Carotid artery plaque (ultrasonography), insulin resistance and liver enzymes (prior to methotrexate (MTX) were determined in 77 consecutive patients with RA (43 with and 34 without MTX).

**Results:**

Serum alanine aminotransferase (ALT) and aspartate aminotransferase (AST) were associated with insulin resistance in univariate analysis (R = 0.54, p < 0.0001 and R = 0.36, p = 0.001, respectively) and after adjustment for age, gender and waist circumference (partial R = 0.43, p = 0.0001 and partial R = 0.37, p = 0.001, respectively). ALT and AST concentrations were higher in patients with plaque as compared to in those without plaque (ALT (u/l): 27 [22-32] versus 20 [18-23], p = 0.02; AST (u/l): 25 [21-28] versus 20 [19-22], p = 0.02). The odds ratios [95% CI] for plaque were 1.92 [1.14–3.24] (p = 0.01), 1.93 [1.17–3.16] (p = 0.009) and 1.82 [1.13–2.93] (p = 0.01) for 1 SD increase in ALT (~10 u/l) and AST (~6 u/l) concentrations and in logarithmically transformed homeostasis model assessment of insulin resistance (HOMA-IR) (~0.2 uU.mmol/ml.l), respectively. After adjustment for the potentially confounding characteristics of age, sex, hypertension and hypothyroidism in logistic regression models, ALT (p = 0.049) and AST concentrations (p = 0.027) remained associated with plaque whereas the HOMA-IR did not (p = 0.08). AST concentrations (p = 0.049) were associated with plaque independent of insulin resistance whereas the HOMA-IR (p = 0.1) was not associated with plaque independent of AST concentrations.

**Conclusion:**

Within currently recommended reference ranges, serum aminotransferase concentrations may be strongly associated with insulin resistance and atherosclerosis in patients with RA. The measurement of aminotransferase concentrations could be a useful tool in cardiovascular risk stratification in patients with RA.

## Background

Insulin resistance is a pathogenetic factor in the metabolic syndrome [1], a constellation of interrelated risk factors that confers a ~2-fold increase in the relative risk for atherosclerotic cardiovascular disease events [2]. Insulin resistance further predicts cardiovascular disease independent of other metabolic syndrome features [3-7]. Rheumatoid arthritis (RA) patients experience a markedly increased prevalence of cardiovascular disease [8-10], a comorbidity that may be partly mediated through insulin resistance [11-15]. Indeed, an independent association of insulin resistance with carotid as well as coronary artery atherosclerosis has been reported in RA [16,17]. However, whereas insulin resistance formed part of the earlier World Health Organization and the European Group for the Study of Insulin Resistance defined metabolic syndrome, the more recent National Cholesterol Education Program's Adult Treatment Panel III (NCEP:APTIII) and the International Diabetes Federation defined metabolic syndrome no longer include insulin resistance for routine assessment of cardiovascular risk [1,18]. The arguments against the use of insulin resistance include its lack of standardization and the ease of measuring other characteristics of the metabolic syndrome [1,18,19]. We and others recently found that in contrast to the homeostasis model assessment of insulin resistance (HOMA-IR) [16,17], the NCEP:ATPIII defined metabolic syndrome was not associated with carotid and coronary artery atherosclerosis in a patients with RA. Therefore, outcome-based studies should be performed to assess the value of HOMA-IR in RA, or an alternative and more sensitive approach should be devised to predict the impact of metabolic syndrome features on cardiovascular risk in RA.

Non-alcoholic fatty liver disease (NAFLD) constitutes a newly recognized integral component of the metabolic syndrome [20,21]. Indeed, magnetic resonance spectroscopy quantified liver fat content is strongly associated with insulin resistance and independent of overall body fat content [22]. Further, elevated aminotransferases (alanine aminotransferase (ALT) and aspartate aminotransferase (AST)) are surrogate markers of liver fat content [20,21] and analysis of the data from the Third National Health and Nutrition Examination Survey revealed a strong association of unexplained aminotransferase elevations with metabolic syndrome features [23]. Whether such relationships exist in patients with RA has not been reported. Importantly, in a 10-year follow-up study, baseline ALT concentrations predicted cardiovascular events [24]. Therefore, since insulin resistance is associated with atherosclerosis whereas serum aminotransferase concentrations are associated with insulin resistance and predict cardiovascular events, it is possible that the respective liver enzymes are equivalent to insulin resistance in their ability to identify subjects with atherosclerosis. If so, serum aminotransferases could be as useful in cardiovascular risk stratification as the assessment of insulin resistance. In the present study, we therefore explored the relationship between serum aminotransferase concentrations and metabolic syndrome features and determined whether serum aminotransferase concentrations are as strongly associated with ultrasonographically located carotid artery atheroma as insulin resistance in RA patients in whom liver function tests would normally be conducted as part of routine assessments.

## Methods

### Patients

We investigated 77 consecutive patients that met the American College of Rheumatology criteria for RA [25]. Their demographics are shown in Table 1. Patients with raised ALT or AST levels (> 35 u/l for ALT and > 34 u/l for AST in women, and > 45 u/l for ALT and > 44 u/l for AST in men, in our laboratory) were excluded if they also had circulating hepatitis B surface antigen or hepatitis C antibody, serological evidence of autoimmune hepatitis (presence of circulating antinuclear, anti smooth-muscle and/or anti liver-kidney microsomal-1 antibodies) or an increased transferrin saturation that may represent hemochromatosis [26]. Other exclusion criteria comprised the use of lipid or glucose lowering agents or medications other than antirheumatic agents that can affect aminotransferase levels. Three patients had diabetes [27] that was managed with dietary intervention only. Since the intake of as little as one alcoholic drink per day in women (83% of our cohort) can increase aminotransferase concentrations [23] and mild and moderate alcohol intake are common in our setting, we did not exclude patients that used alcohol but controlled for alcohol consumption in our analyses. Also, we did not exclude patients with hypothyroidism when they were euthyroid on replacement therapy at the time of the study. Informed consent was obtained from each patient and the study was approved by The Ethics Committee for Research on Human Subjects (Medical) of the University of the Witwatersrand.

**Table 1:** Baseline characteristics in 77 rheumatoid arthritis patients. Values are mean [95% CI] or n (%).

**Characteristic**	All patients (n = 77)	Patients with plaque (n = 25)	Patients without plaque (n = 52)	p^†^
**Age (years)**	54 [51–56]	60 [57–63]	51 [48–54]	0.0002
**Women**	64 (83)	21 (84)	43 (83)	0.9
**Caucasian: Asian**	69 (90): 8 (10)	23 (92): 2 (8)	46 (88): 6 (12)	0.6
**Life style factors**				
Current smokers	17 (22)	8 (32)	9 (17)	0.2
Current smoking (cigarettes/day)*	0.8 [0.4–1.4]	1.5 [0.4–3.3]	0.6 [0.2–1.2]	0.2
Current alcohol users	26 (34)	9 (36)	17 (33)	0.8
Current alcohol (units/week)*	1 [0.6–1.6]	1.2 [0.4–2.6]	1.0 [0.4–1.7]	0.7
Current exercisers	22 (29)	6 (25)	16 (31)	0.6
Exercise (hours/week)*	0.5 [0.3–0.8]	0.4 [0.1–0.9]	0.6 [0.3–0.9]	0.6
Disease duration (years)*	6.6 [4.9–8.7]	9.2 [5.6–15.2]	5.6 [4.0–7.8]	0.1
**Rheumatoid factor positive**	61 (79)	19 (76)	42 (81)	0.6
**Acute phase reactants**				
Hs-C-reactive protein (mg/l)*	7.4 [5.4–10.2]	9.4 [5.8–15.2]	6.7 [4.3–10.2]	0.3
ESR (mm/hr)*	14 [10–18]	11 [7–17]	15 [10–21]	0.3
**Other cardiovascular risk factors**				
Waist circumference (cm)	86 [83–88]	88 [83–93]	85 [81–88]	0.3
Body mass index (kg/m^2^)	24.4 [23.4–25.3]	24.2 [22.8–26.0]	24.3 [23.1–25.6]	0.9
Hypertension	34 (44)	19 (76)	15 (29)	0.0002
Systolic blood pressure (mmHg)	127 [124–131]	131 [124–138]	125 [121–129]	0.1
Diastolic blood pressure (mmHg)	82 [80–84]	81 [78–85]	82 [80–85]	0.6
Total cholesterol (mmol/l)	5.1 [4.9–5.3]	5.0 [4.6–5.3]	5.1 [4.9–5.4]	0.5
LDL cholesterol (mmol/l)	2.9 [2.7–3.0]	2.8 [2.5–3.1]	2.9 [2.7–3.1]	0.5
HDL cholesterol (mmol/l)	1.6 [1.5–1.7]	1.7 [1.5–1.9]	1.6 [1.5–1.7]	0.5
Triglycerides (mmol/l)*	1.1 [1.0–1.2]	1.2 [1.0–1.4]	1.0 [0.9–1.1]	0.08
HOMA-IR (uU.mmol/ml.l)*	1.15 [1.02–1.29]	1.45 [1.18–1.77]	1.03 [0.90–1.19]	0.009
Glucose (mmol/l)	4.3 [4.2–4.5]	4.3 [4.2–4.5]	4.5 [4.2–4.5]	0.8
Diabetes	3 (4)	0 (0)	3 (6)	0.3
Hypothyroidism	19 (25)	10 (40)	9 (17)	0.03
Framingham score*	4 [4–5]	6 [5–8]	4 [3–5]	0.009
**CCA intima-media thickness (mm)**	0.65 [0.63–0.68]	0.73 [0.69–0.78]	0.62 [0.60–0.64]	0.0001

CI, confidence interval; Hs, high sensitivity; ESR, erythrocyte sedimentation rate; LDL, low density lipoprotein; HDL, high density lipoprotein; HOMA-IR, homeostasis model assessment of insulin resistance; CCA, common carotid artery.

*Variables for which geometric means [95% CI] are given. ^†^For comparison of characteristics between patients with plaque and patients without plaque.

### Clinical and laboratory data and carotid ultrasonography

These data (Tables 1 and 2) were obtained through patient interview, physical examination, medical record review and laboratory tests performed on fasting blood samples. We recorded life style factors, RA characteristics and other traditional and nontraditional risk factors (Table 1). Hypertension was diagnosed in patients with a blood pressure of ≥140/90 mmHg and in those employing antihypertensive agents. We measured high-sensitivity C-reactive protein and lipid levels, plasma glucose, serum insulin and thyrotropin concentrations [28]. Insulin resistance was estimated by the homeostasis model assessment of insulin resistance (HOMA-IR) using the formula = (insulin (uU/ml) × glucose (mmol/l)) ÷ 22.5 [29]. Diabetes was diagnosed in accordance with the recent recommendations as made by the American Diabetes Association [27].

**Table 2:** Antirheumatic agents and aminotransferase concentrations in 77 rheumatoid arthritis patients

**Characteristic**	Mean [95% CI] or n (%)
**Antirheumatic agents in 34 methotrexate naïve patients**	
Non-steroidal antiinflammatory agents	14 (41)
Prednisone users	4 (12)
Prednisone dose (mg/l)*	1.2 [1.0–1.4]
Disease modifying agents	
Users	13 (38)
Leflunomide	6 (18)
Chloroquine	5 (15)
Sulphasalazine	3 (9)
Azathioprine	2 (6)
**Antirheumatic agents in 43 patients before methotrexate use**	
Non-steroidal antiinflammatory agents	32 (74)
Prednisone users	17 (40)
Prednisone dose (mg/l)*	2.4 [1.7–3.4]
Disease modifying agents	
Users	13 (30)
Sulphasalazine	10 (23)
Chloroquine	3 (7)
**Antirheumatic agents in 43 patients using methotrexate**	
Non-steroidal antiinflammatory agents	20 (48)
Prednisone users	7 (16)
Prednisone dose (mg/l)*	1.0 [1.1–1.5]
Disease modifying agents	
Methotrexate dose (mg/week)	19.6 [17.5–21.7]
Chloroquine	21 (49)
Minocyclin	12 (28)
Leflunomide	5 (12)
Sulphasalazine	4 (9)
Azathioprine	3 (7)
**Aminotransferase levels in 34 methotrexate naïve patients**	
Alanine aminotransferase (u/l)	27 [22–31]
Aspartate aminotransferase (u/l)	23 [22–25]
**Aminotransferase levels in 43 patients before methotrexate use**	
Alanine aminotransferase (u/l)	19 [17–22]
Aspartate aminotransferase (u/l)	20 [18–22]
**Aminotransferase levels in 43 patients using methotrexate**	
Alanine aminotransferase (u/l)	38 [33–42]
Aspartate aminotransferase (u/l)	30 [27–34]
**Increases in aminotransferase levels in 43 patients on methotrexate**	
Alanine aminotransferase (u/l)	18 [14–23]^†^
Aspartate aminotransferase (u/l)	11 [7–14]^†^
**Change in weight in 43 patients since using methotrexate (kg)**	-0.7 [-0.2–0.6]^‡^
**Duration of methotrexate therapy in 43 patients (years)**	5.1 [4.1–6.1]

*Variables for which geometric means [95% CI] are given. ^†^p < 0.0001; ^‡^p = 0.7.

Carotid artery plaque was defined as a localized irregular intima-media thickening of ≥1.5 mm as identified by high resolution B-mode ultrasonography (Image Point, Hewlett Packard Andover, MA, USA). We also measured the common carotid artery intima-media thickness more than 1 cm proximal to the common carotid artery flow divider. All ultrasonographic evaluations were performed by the same investigator who was unaware of the CV risk factor profiles of the patients. The coefficient of variation for the intraobserver variability was 5.7% and further details of carotid artery ultrasonography in our unit have been previously reported on [28].

ALT and AST concentrations were determined using the α-ketoglutarate reaction (Abbott Laboratories, Diagnostic Division, Abbott Park, Illinois 60064). Of the 77 patients, 34 were methotrexate naïve and 43 were using methotrexate (Table 2). ALT and AST concentrations were measured in all patients at the time that carotid artery ultrasonography was performed. Additionally, in patients using methotrexate, aminotransferase concentrations had been recorded immediately prior to methotrexate initiation and, in the respective patients, these levels were used in the main analyses of this report. Since weight loss and weight gain are both associated with changes in aminotransferase concentrations and dietary advice is routinely provided in our setting [15], we also determined changes in weight during methotrexate therapy. Finally, drug therapy that was used prior to methotrexate initiation was also recorded.

### Statistical analysis

Results were expressed as mean [95% confidence interval (CI)] or number (%) unless indicated otherwise. For non-normally distributed data, the geometric means [95% CI] are given and these data were also logarithmically transformed prior to statistical analysis.

We compared baseline recorded characteristics in patients with and without plaque using the Student's t test for continuous variables and the chi-square test or the Fisher's exact test for categorical variables.

Associations between aminotransferase levels and metabolic syndrome features were assessed in simple and mixed regression models or by the Student's t test and in logistic regression models, as appropriate. Whether aminotransferase concentrations contribute to the variability in insulin resistance independent of abdominal obesity was also determined in mixed regression models. Because many univariate analyses were performed, we verified univariate associations in multivariable regression models with consideration of interactions, collinearity and causality in which significance was set at 0.05.

The associations between aminotransferase concentrations and plaque were assessed by the Student's t test and subsequently in logistic regression models with entering of a one standard deviation (SD) increase in ALT or AST concentrations with or without potentially confounding characteristics, as independent variables in the model. Similar models were built to assess the associations of HOMA-IR and waist circumference with plaque. Since 25 patients had plaque, we took into account that entering a large number of independent variables in logistic regression models may constitute overfitting [30].

In studies performed on large numbers of RA patients, carotid intima-media thickness was shown to be associated with an extensive number of cardiovascular risk factors [31,32]. In view of our small sample size, we did not systematically assess the association of cardiovascular risk factors with common carotid artery intima-media thickness.

## Results

### Descriptive data

Descriptive data are shown in Tables 1 and 2. Of note, 26 (34%) patients consumed alcohol (Table 1). The number of units of alcohol used per week in these patients was 1 in 2, 2 in 3, 3 in 2, 4 in 3, 6 in 2, 7 in 2, 8 in 1, 10 in 2, 11 in 1, 14 in 2, 20 in 1, 21 in 2, 26 in 1, 28 in 1 and 35 in 1 patient, respectively. In keeping with previous reports by us [16,28], plaque was associated with age (p = 0.0002), hypertension (p = 0.0002), the HOMA-IR (p = 0.0009), hypothyroidism (p = 0.03) and the Framingham score (p = 0.009). The mean common carotid artery intima-media was 0.13 mm thicker in patients with plaque as compared to in those without plaque (p = 0.0001). While not on methotrexate, raised ALT and AST concentrations were present in 5 patients and 1 patient, respectively, and the highest ALT value was 63 u/l whereas that of AST was 40 u/l (Table 2). Upon the use of methotrexate, there was no change in body weight whereas serum ALT concentrations increased by 18 [14-23] u/l and AST concentrations by 11 [7-14] u/l (Table 2). Of the 43 patients on methotrexate, 16 experienced increased ALT levels and 6 increased AST levels, and the highest ALT value was 83 u/l whereas that of AST was 61 u/l. All 43 patients on methotrexate were using folic acid and in them, aminotransferase levels that were used in the main analysis (see methods) had been measured 5.1 [4.1–6.1] years previously (see duration of methotrexate therapy in Table 2).

### Relationship between aminotransferase concentrations and metabolic syndrome features

Associations between aminotransferase concentrations (those measured at the time that carotid artery ultrasonography was performed in methotrexate naïve patients and those measured immediately prior to methotrexate therapy in the other patients (see methods)) and metabolic syndrome features are shown in Table 3. ALT concentrations were associated with insulin resistance, triglyceride levels and waist circumference. AST concentrations were also associated with insulin resistance and triglyceride levels but not with waist circumference. Neither ALT nor AST concentrations were associated with glucose and HDL cholesterol levels and systolic and diastolic blood pressure. Aminotransferase levels were also not associated with a diagnosis of hypertension (ALT = 25 [21-29] u/l in hypertensives and 21 [18-24] u/l in normotensives (p = 0.10); AST = 23 [20-26] u/l in hypertensives and 21 [19-23] in normotensives (p = 0.13)). With regard to potential confounding characteristics in this context, aminotransferase concentrations were not associated with the use of non-steroidal antiinflammatory agents (ALT = 21 [18-23] u/l in users (n = 46 (60%)) and 23 [20-27] u/l in non-users (p = 0.4); AST = 21 [19-23] u/l in users and 22 [20-24] u/l in non-users (p = 0.4)) and with the use of leflunomide (ALT = 25 [21-30] u/l in users (n = 6 (8%)) and ALT = 22 [19-25] u/l in non-users (p = 0.2); AST = 23 [19-27] u/l in users and AST = 21 [20-23] u/l in non-users (p = 0.3)). In separate multivariable models in which the use of non-steroidal antiinflammatory agents or leflunomide were adjusted for, ALT concentrations remained associated with the log HOMA-IR (p < 0.0001), waist circumference (p ≤ 0.0003) and log triglycerides (p ≤ 0.0002), and AST concentrations remained associated with log HOMA-IR (p ≤ 0.002) and log triglycerides (p ≤ 0.001). Other potential confounding characteristics in this context comprise alcohol use, changes in weight on methotrexate, age, gender, use of glucocorticoids, minocyclin, sulphasalazine and azathioprine, systemic inflammation as reflected by C-reactive protein concentrations and the erythrocyte sedimentation rate, and hypothyroidism [20,33-37]. None of these variables were associated with aminotransferase concentrations (p ≥ 0.1). In separate multivariable models in which these characteristics were adjusted for, ALT remained associated with log HOMA-IR (p < 0.0001), waist circumference (p ≤ 0.0007) and log triglycerides (p ≤ 0.0004), and AST concentrations remained associated with log HOMA-IR (p ≤ 0.002) and log triglycerides (p ≤ 0.002).

**Table 3:** Associations between aminotransferase concentrations and metabolic Syndrome features

**Metabolic syndrome features**	ALT (u/l)		AST (u/l)	
		
	R	p	R	p
Log HOMA-IR (uU.mmol/ml.l)	0.54	< 0.0001	0.36	0.001
Glucose (mmol/l)	0.20	0.09	0.11	0.4
Waist circumference (cm)	0.41	0.0002	0.09	0.4
Log triglycerides (mmol/l)	0.42	0.0002	0.37	0.001
HDL cholesterol (mmol/l)	-0.12	0.3	0.01	0.9
Systolic blood pressure (mmHg)	0.01	0.9	-0.05	0.7
Diastolic blood pressure (mmHg)	0.07	0.5	-0.03	0.8

ALT, alanine aminotransferase; AST, aspartate aminotransferase; Log, logarithmically transformed; HOMA-IR, homeostasis model assessment of insulin resistance.

ALT concentrations while taking methotrexate were still associated the HOMA-IR (R = 0.34, p = 0.03) but no longer with other metabolic syndrome features (p ≥ 0.09). AST concentrations on methotrexate were no longer associated with any of the metabolic syndrome features (p ≥ 0.09).

### The relative contribution of aminotransferase concentrations and abdominal adiposity to the variability in insulin resistance

In mixed regression models in which age and sex were adjusted for, ALT and AST concentrations contributed to the HOMA-IR independent of waist circumference (Table 4). The standardized regression coefficient (SE) for the independent relationships between ALT concentrations and insulin resistance and between AST concentrations and insulin resistance were as large as that for the relationship between abdominal adiposity and insulin resistance (p ≥ 0.2) thereby indicating that aminotransferase concentrations and abdominal adiposity contributed to the variability of insulin resistance to a similar extent. ALT and AST concentrations also contributed to the HOMA-IR independent of the body mass index (p < 0.0001 and = 0.001, respectively).

**Table 4:** Mixed regression models for insulin resistance (log HOMA-IR)

	Model 1 (R^2 ^= 0.40)				Model 2 (R^2 ^= 0.37)			
		
	ß ± SE	stand ß ± SE	pR	*P*	ß ± SE	Stand ß ± SE	pR	*P*
Waist ÷ 10 (cm)	0.06 ± 0.02	0.29 ± 0.09	0.31	0.007	0.08 ± 0.02	0.42 ± 0.10	0.45	0.007
ALT ÷ 10 (u/l)	0.09 ± 0.02	0.41 ± 0.10	0.43	0.0001				
AST÷ 10 (u/l)					0.12 ± 0.03	0.32 ± 0.09	0.37	0.001

Age and gender were entered as covariates in the models.

HOMA-IR, homeostasis model assessment of insulin resistance; ß ± SE, regression coefficient ± standard error; stand, standardized; pR, partial correlation coefficient; ALT, alanine aminotransferase; AST, aspartate aminotransferase.

ALT and AST concentrations while taking methotrexate were not significantly associated with insulin resistance independent of abdominal adiposity (partial R = 0.30; p = 0.06 and partial R = 0.28; p = 0.08, respectively). These associations may not have been significant because of the small sample size in which this analysis was performed, i.e. only 43 patients were using methotrexate.

### Relationships between aminotransferase concentrations and carotid artery atherosclerosis

**Figure 1:**
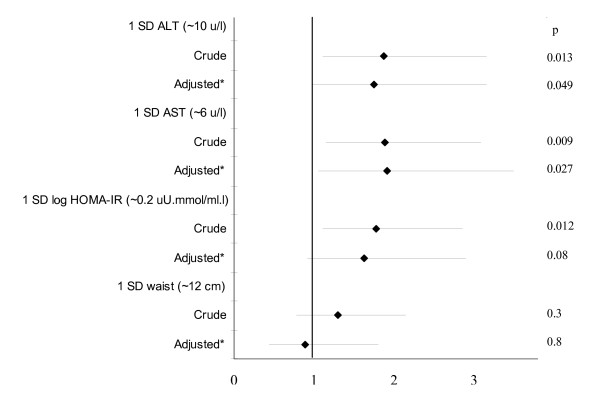
Associations of aminotransferases, HOMA-IR and waist circumference with ultrasonographically located carotid artery plaque in logistic regression models. *Adjusted for age, gender, hypertension and hypothyroidism. SD, standard deviation; ALT, alanine aminotransferase; AST, aspartate aminotransferase; HOMA-IR, homeostasis model assessment of insulin resistance; CI, confidence interval.

ALT and AST concentrations were higher in patients with plaque as compared to in those without plaque (ALT (u/l): 27 [22-32] vs 20 [18-23], p = 0.02; AST (u/l): 25 [21-28] vs 20 [19-22], p = 0.02). Using the 95% CI values of ALT (23 u/l) and AST (22 u/l) in patients without plaque as threshold values, having a high ALT or/and AST concentration (n = 41 (53%)) was associated with plaque (odds ratio (OR) [95% CI] = 4.3 [1.5–12.8], p = 0.007). The associations between aminotransferase concentrations and carotid artery plaque were further assessed in logistic regression models as illustrated in Figure 1. The OR [95% CI] for plaque was 1.92 [1.14–3.24] (p = 0.01) and 1.93 [1.17–3.16] (p = 0.009) for 1 SD increase in ALT (~10 u/l) and AST concentrations (~6 u/l), respectively. Adjustments for age, gender, hypertension and hypothyroidism did not materially affect the associations of aminotransferase concentrations with carotid artery plaque (Figure 1). After adjustment for the Framingham score, ALT and AST concentrations also remained associated with plaque (OR [95% CI] = 1.92 [1.14–3.23] (p = 0.01) and 2.05 [1.22–3.43] (p = 0.006) for 1 SD increase in ALT and AST, respectively). Further, adjustment for alcohol intake and changes in weight on MTX did not substantially affect the associations of aminotransferase concentrations with common carotid artery plaque (p = 0.03 for ALT and p = 0.02 for AST). After adjusting for insulin resistance, AST concentrations remained associated with plaque (p = 0.049). Aminotransferase concentrations were not associated with CCA-IMT (R = 0.19, p = 0.1 for ALT and R = 0.09, p = 0.4 for AST).

Aminotransferase concentrations in patients while taking methotrexate were not associated with plaque (p = 0.2 for both ALT and AST).

Abdominal adiposity and insulin resistance reportedly constitute etiopathogenetic mechanisms in the metabolic syndrome [1,2,19]. As further shown in Figure 1 and as compared to aminotransferase concentrations, waist circumference was not associated with plaque whereas the HOMA-IR was associated with plaque in univariate analysis but no longer (p = 0.08) after age, gender, hypertension and hypothyroidism were controlled for. After adjustment for the Framingham score, the HOMA-IR was associated with plaque (OR [95% CI] = 1.80 [1.12–2.91] (p = 0.02) for 1 SD increase in HOMA-IR) whereas waist circumference was not associated with plaque (OR [95% CI] = 1.29 [0.77–2.17] (p = 0.3) for 1 SD increase in waist circumference). Adjusted for alcohol intake and changes in weight on MTX did not materially affect these associations (p = 0.02 for HOMA-IR and p = 0.3 for waist circumference). Further, after adjustment for ALT or AST concentrations, the HOMA-IR was no longer associated with plaque (p ≥ 0.1).

Disease activity and inflammation enhance cardiovascular risk in RA [31,32]. In this cohort, C-reactive protein concentrations were not associated with carotid artery intima-media thickness (R = 0.08, p = 0.5) or plaque (C-reactive protein = 9.4 [5.8–15.5] mg/dl in those with plaque and 6.7 [4.4–10.2] mg/dl in those without plaque (p = 0.3)). This may be related to the fact that only one measurement of C-reactive protein in time was considered and to our small sample size. Also, after adjustment for C-reactive protein concentrations, ALT, AST and the HOMA-IR remained associated with plaque (OR [95% CI] = 1.87 [1.11–3.18] (p = 0.02), 1.89 [1.15–3.11] (p = 0.01) and 1.80 [1.12–2.89] (p = 0.01) for 1 SD increase in ALT, AST and HOMA-IR, respectively).

## Discussion

In this study we found, for the first time, that high serum aminotransferase concentrations were associated with the presence of carotid artery plaque independent of age, gender, hypertension and hypothyroidism, and of conventional cardiovascular risk factors as estimated by the Framingham score. Whereas NAFLD is a potentially newly identified pathogenetic mechanism in the metabolic syndrome [21,38] and cardiovascular disease, abdominal adiposity and insulin resistance are the most recognized pathogenetic pathways in the metabolic syndrome [1,2,19]. We found no association between waist circumference and plaque. This finding is in keeping with previous reports on cardiovascular disease in RA [16,17] and argues against the use of waist circumference as an alternative to insulin resistance in the assessment of cardiovascular risk in this disease. Importantly, in univariate analysis, the odds ratios for plaque were very similar at 1.92, 1.93 and 1.82 for 1 SD increase in ALT (~10 u/l) and AST concentrations (~6 u/l) and in HOMA-IR (~0.2 uU.mmol/ml.l), respectively, with extensively overlapping 5 to 95% confidence intervals for these 3 metabolic parameters. Moreover, whereas AST concentrations were associated with plaque independent of insulin resistance, the HOMA-IR was not associated with plaque independent of AST concentrations. Taken together, serum aminotransferase concentrations performed at least as well as insulin resistance as assessed by the HOMA-IR in the identification of the presence of carotid artery atherosclerosis in this RA cohort.

In our analysis, aminotransferase concentrations contributed as much as waist circumference to the variability in insulin resistance. This suggests that the determination of aminotransferase concentrations can make a valuable additional contribution to waist circumference measurements in the identification of subjects with insulin resistance, a risk factor not only for cardiovascular disease but also for type 2 diabetes mellitus [1,2,19]. Both directly estimated liver fat content and high aminotransferase concentrations are reportedly strongly associated with metabolic syndrome features in individuals without RA [21-23]. In the present cohort, a consistent relationship between aminotransferase concentrations and triglycerides and the HOMA-IR was found. AST concentrations may reflect liver fat content less reliably than ALT levels since liver fibrosis can also contribute to high AST concentrations [20]. ALT concentrations were associated with waist circumference whereas AST concentrations were not.

Our finding of an association of aminotransferase concentrations with metabolic syndrome features suggests that high aminotransferases are caused by NAFLD [23]. We considered an extensive range of potentially confounding variables including used medications, in our analyses. Once other causes of chronic liver disease are excluded, non-alcoholic fatty liver disease is the cause of asymptomatic elevations of aminotransferase levels in 90% of cases [20] and magnetic resonance spectroscopy quantified liver fat content correlates with ALT concentrations in non-RA subjects (r = 0.4 to 0.6, p < 0.001) [22]. However, we did not directly estimate liver fat content and, therefore, whether high aminotransferase concentrations reflect NAFLD in patients with RA as applies to non-RA subjects, needs to be determined.

The use of current metabolic syndrome definitions and even the existence of the metabolic syndrome have recently been questioned [40]. This issue has also been addressed in previously RA studies [16,17]. These reports together with our current findings argue for considering both insulin resistance and aminotransferase concentrations as metabolic syndrome features in cardiovascular risk stratification in RA. Notably, these characteristics do not form part of the NCEP: ATP III and International Diabetes Federation defined metabolic syndrome [1,18].

We found that mean ALT and AST levels were 27 u/l and 25 u/l in patients with plaque, respectively. The corresponding values for ALT and AST in patients without plaque were each 20 u/l. Further, levels of > 23 u/l for ALT and 22 u/l for AST (95% CI values in patients without plaque) were associated with a 4-fold increased prevalence of plaque. Thus, methotrexate naïve RA patients with ALT concentrations > 23 u/l or/and AST concentrations > 22 u/l were at high risk of having severe atherosclerosis, thereby suggesting that RA patients with aminotransferase levels above these cut points require further evaluation for the presence of atherosclerosis. Such patients may further benefit from interventions that are recommended in NAFLD [20-24]. These aspects need to be addressed in future larger studies. Such levels of aminotransferases are well within normal reference ranges used in our laboratory. Indeed, Prati and colleagues [39] reported that most patients with non-alcoholic fatty liver disease or chronic asymptomatic hepatitis C virus infection have ALT concentrations that are within currently recommended reference ranges, and showed that a reduction of upper limits for ALT to 19 u/l in women and 30 u/l in men increased the sensitivity in identifying hepatitis C viremia from 55% to 76% with an acceptable reduction in specificity from 97% to 89%.

We investigated a cohort of RA patients. Since the prevalence of both cardiovascular disease and insulin resistance is increased in RA [8-15], our results need to be confirmed in subjects without RA. Whereas methotrexate use in RA is associated with both a marked reduction in cardiovascular mortality [41] and increased aminotransferase concentrations, in contrast to our findings prior to methotrexate therapy, we no longer found an association between aminotransferase levels and atherosclerosis once methotrexate had been initiated. Our findings also do not support the use of aminotransferase concentrations in the identification of elevated cardiovascular risk once on methotrexate. We did not assess the hepatitis B surface antigen and hepatitis C antibody status in patients with ALT or AST levels below normal reference ranges as used in our laboratory. However, both hepatitis B and C infection occur infrequently in South African Caucasians and Asians [42]. We measured insulin resistance using the HOMA-IR rather than the hyperinsulinemic euglycemic clamp technique, and abdominal obesity was estimated by waist circumference and not by computed tomography or magnetic resonance imaging. Conversely, characteristics as recorded in this study are often accessible in routine clinical care settings. As in investigations previously reported by us, we defined plaque as a localized irregular intima-media thickening of ≥1.5 mm [16,28,43,44] whereas others considered plaque to be present when a ≥50% protrusion beyond the diameter of the surrounding wall was found (eg. Roman et al [45]). The mean common carotid artery intima-media thickness was 0.73 mm in our patients with plaque. In a RA cohort of 91 subjects, we recently identified 6 cardiovascular risk factors that were independently associated with plaque [44]. This compares favorably to the study of Roman et al in which only 3 independent risk factors for atherosclerosis were identified in 98 RA patients [45]. The optimal criteria for defining the presence of plaque in RA deserve further study in larger cohorts. Finally, one could argue that it would be more straightforward to perform imaging studies since liver enzymes are not directly involved in atherogenesis. However, in contrast to the determination of aminotransferase concentrations, carotid ultrasonography is currently not routinely performed in patients with RA.

## Conclusion

Our findings suggest that, within currently recommended reference ranges, high aminotransferase concentrations may be strongly associated with insulin resistance in RA. Moreover, such aminotransferase elevations were closely associated with atherosclerosis. Whether determination of aminotransferase concentrations is a useful tool in the assessment of cardiovascular risk in patients with RA awaits replication of our results in other settings and in different populations with larger sample sizes.

## Competing interests

The author(s) declare that they have no competing interests.

## Authors' contributions

PHD conceived and designed the study, acquired the data and performed the initial analysis and interpretation of the data and draft of the manuscript. GRN and AJW contributed substantially to the analysis and interpretation of the data as well as to the drafting and revision of the manuscript. BIJ contributed substantially to the interpretation of the data and revision of the manuscript. All authors gave final approval of the version to be published.

## Pre-publication history

The pre-publication history for this paper can be accessed here:



## References

[B1] Eckel RH, Grundy SM, Zimmet PZ (2005). The metabolic syndrome. Lancet.

[B2] Grundy SM, Cleeman JI, Daniels SR, Donato KA, Eckel RH, Franklin BA, Gordon DJ, Krauss RM, Savage PJ, Smith SC, Spertus JA, Costa F, American Heart Association, National Heart, Lung, and Blood Institute (2005). Diagnosis and management of the metabolic syndrome. An American Heart Association/National Heart, Lung, and Blood Institute Scientific Statement. Executive Summary. Cardiol Rev.

[B3] Yip J, Facchini FS, Reaven GM (1998). Resistance to insulin-mediated glucose disposal as a predictor of cardiovascular disease. J Clin Endocrinol Metab.

[B4] Hanley AJ, Williams K, Stern MP, Haffner SM (2002). Homeostasis model assessment of insulin resistance in relation to the incidence of cardiovascular disease: the San Antonio Heart Study. Diabetes Care.

[B5] Hedblad B, Nilsson P, Engstrom G, Berglund G, Janzon L (2007). Insulin resistance in non-diabetic subjects is associated with increased incidence of myocardial infarction and death. Diabet Med.

[B6] Bonora E, Kiechl S, Willeit J, Oberhollenzer F, Egger G, Meigs JB, Bonadonna RC, Muggeo M (2007). Insulin resistance as estimated by homeostasis model assessment predicts incident symptomatic cardiovascular disease in Caucasian subjects from the general population. Diabetes Care.

[B7] Reilly MP, Wolfe ML, Rhodes T, Girman C, Mehta N, Rader DJ (2004). Measures of insulin resistance add incremental value to the clinical diagnosis of metabolic syndrome in association with coronary atherosclerosis. Circulation.

[B8] Solomon DH, Karlson EW, Rimm EB, Cannuscio CC, Mandl LA, Manson JE, Stampfer MJ, Curhan GC (2003). Cardiovascular morbidity and mortality in women diagnosed with rheumatoid arthritis. Circulation.

[B9] Maradit-Kremers H, Nicola PJ, Crowson CS, Ballman KV, Gabriel SE (2005). Cardiovascular death in rheumatoid arthritis: a population-based study. Arthritis Rheum.

[B10] Goodson N, Marks J, Lunt M, Symmons D (2005). Cardiovascular admissions and mortality in an inception cohort of patients with rheumatoid arthritis with onset in the 1980s and 1990s. Ann Rheum Dis.

[B11] Sattar N, McCarey DW, Capell H, McInnes IB (2003). Explaining how "high-grade" systemic inflammation accelerates vascular risk in rheumatoid arthritis. Circulation.

[B12] Gonzalez-Gay MA, Gonzalez-Juanatey C, Martin J (2005). Rheumatoid arthritis: a disease associated with accelerated atherogenesis. Semin Arthritis Rheum.

[B13] Gonzalez-Gay MA, De Matias JM, Gonzalez-Juanatey C, Garcia-Porrua C, Sanchez-Andrade A, Martin J, Llorca J (2006). Anti-tumor necrosis factor-alpha blockade improves insulin resistance in patients with rheumatoid arthritis. Clin Exp Rheumatol.

[B14] Dessein PH, Stanwix AE, Joffe BI (2002). Cardiovascular risk in rheumatoid arthritis versus osteoarthritis: acute phase response related decreased insulin sensitivity and high-density lipoprotein cholesterol as well as clustering of metabolic syndrome features in rheumatoid arthritis. Arthritis Res.

[B15] Dessein PH, Joffe BI, Stanwix AE (2002). Effects of disease modifying agents and dietary intervention on insulin resistance and dyslipidemia in inflammatory arthritis: a pilot study. Arthritis Res.

[B16] Dessein PH, Tobias M, Veller MG (2006). Metabolic syndrome and subclinical atherosclerosis in rheumatoid arthritis. J Rheumatol.

[B17] Chung CP, Oeser A, Solus JF, Avalos I, Gebretsadik T, Shintani A, Raggi P, Sokka T, Pincus T, Stein CM (2007). Prevalence of the metabolic syndrome is increased in rheumatoid arthritis and is associated with coronary atherosclerosis. Atherosclerosis.

[B18] Alberti KGMM, Zimmet P, Shaw J (2005). The metabolic syndrome-a new worldwide definition. Lancet.

[B19] Grundy SM, Cleeman JI, Daniels SR, Donato KA, Eckel RH, Franklin BA, Gordon DJ, Krauss RM, Savage PJ, Smith SC, Spertus JA, Costa F (2005). Diagnosis and management of the metabolic syndrome: An American Heart Association/National Heart, Lung, and Blood Institute Scientific Statement. Circulation.

[B20] Angulo P (2002). Nonalcoholic fatty liver disease. N Engl J Med.

[B21] Yki-Jarvinen H (2005). Fat in the liver and insulin resistance. Ann Med.

[B22] Westerbacka J, Corner A, Tiikkainen M, Tamminen M, Vehkavaara S, Hakkinen AM, Fredriksson J, Yki-Jarvinen H (2004). Women and men have similar amounts of liver and intra-abdominal fat, despite more subcutaneous fat in women: implications for sex differences in markers of cardiovascular risk. Diabetologia.

[B23] Clark JM, Brancati FL, Diehl AM (2003). The prevalence and etiology of elevated aminotransferase levels in the United States. Am J Gastroenterol.

[B24] Schindhelm RK, Dekker JM, Nijpels G, Bouter LM, Stehouwer CDA, Heine RJ, Diamant M (2007). Alanine aminotransferase predicts coronary heart disease events: a 10-year follow-up of the Hoorn Study. Atherosclerosis.

[B25] Arnett FC, Edworthy SM, Bloch DA, McShane DJ, Fries JF, Cooper NS (1988). The American Rheumatology Association 1987 revised criteria for the classification of rheumatoid arthritis. Arthritis Rheum.

[B26] Andrews NC (1999). Disorders of iron metabolism. N Engl J Med.

[B27] American Diabetes Association (2005). Diagnosis and classification of diabetes mellitus. Diabetes Care.

[B28] Dessein PH, Joffe BI, Veller MG, Stevens BA, Tobias M, Reddy K, Stanwix AE (2005). Traditional and nontraditional cardiovascular risk factors are associated with atherosclerosis in rheumatoid arthritis. J Rheumatol.

[B29] Wallace TM, Levy JC, Matthews DR (2004). Use and abuse of HOMA modeling. Diabetes Care.

[B30] Peduzzi P, Concato J, Kemper E, Holford TR, Feinstein AR (1996). A simulation study of the number of events per variable in logistic regression analysis. J Clin Epidemiol.

[B31] del Rincon I, Williams K, Stern MP, Freeman GL, O'Leary DH, Escalante A (2003). Association between carotid atherosclerosis and markers of inflammation in rheumatoid arthritis patients and healthy subjects. Arthritis Rheum.

[B32] del Rincon I, Freeman GL, Haas RW, O'Leary DH, Escalante A (2005). Relative contribution of cardiovascular risk factors and rheumatoid arthritis clinical manifestations to atherosclerosis. Arthritis Rheum.

[B33] Siest G (2004). Study of reference values and biological variation: a necessity and a model for Preventive Medicine Centers. Clin Chem Lab Med.

[B34] Saha B, Maity C (2002). Alteration of serum enzymes in primary hypothyroidism. Clin Chem Lab Med.

[B35] Chang Y, Ryu S, Sung E, Jang Y (2007). Higher concentrations of alanine aminotransferase within the reference interval predict nonalcoholic fatty liver disease. Clin Chem.

[B36] Kerner A, Avizohar O, Sella R, Bartha P, Zinder O, Markiewicz W, Levy Y, Brook GJ, Aronson D (2005). Association between elevated liver enzymes and C-reactive protein: possible hepatic contribution to systemic inflammation in the metabolic syndrome. Arterioscler Thromb Vasc Biol.

[B37] Yamada J, Tomiyama H, Yambe M, Koji Y, Motobe K, Shiina K, Yamamoto Y, Yamashina A (2006). Elevated serum levels of alanine aminotransferase and gamma glutamyltransferase are markers of inflammation and oxidative stress independent of the metabolic syndrome. Atherosclerosis.

[B38] den Boer M, Voshol PJ, Kuipers F, Havekes LM, Romijn JA (2004). Hepatic steatosis: a mediator of the metabolic syndrome. Lessons from animal models. Arterioscler Thromb Vasc Biol.

[B39] Prati D, Taioli E, Zanella A, Della Torre E, Butelli S, Del Vecchio E, Vianella L, Zanuso F, Mozzi F, Milani S, Conte D, Colombo M, Sirchia G (2002). Updated definitions of healthy ranges for serum alanine aminotransferase levels. Ann Intern Med.

[B40] Kahn R, Buse J, Ferrannini E, Stern M (2005). American Diabetes Association: European Association for the Study of Diabetes. The metabolic syndrome: time for a critical appraisal. Diabetes Care.

[B41] Choi HK, Heman MA, Seeger JD, Robins JM, Wolfe F (2002). Methotrexate and mortality in patients with rheumatoid arthritis: a prospective study. Lancet.

[B42] Ellis LA, Brown D, Conradie JD, Paterson A, Sher R, Millo J, Theodossiadou E, Dusheiko GM (1990). Prevalence of hepatitis C in South Africa: detection of anti-HCV in recent and stored serum. J Med Virol.

[B43] Veller MG, Fisher CM, Nicolaides AN, Renton S, Geroulakos G, Stafford MJ, Sarker A, Szendro G, Belcaro G (1993). Measurement of the ultrasonic intima-media complex thickness in normal subjects. J Vasc Surg.

[B44] Dessein PH, Norton GR, Woodiwiss AJ, Joffe BI, Wolfe F (2007). Impact of non-classical cardiovascular risk factors on the accuracy of predicting subclinical atherosclerosis in rheumatoid arthritis. J Rheumatol.

[B45] Roman MJ, Moeller E, Davis A, Paget SA, Crow MK, Lockshin MD, Sammaritano L, Devereux RB, Schwartz JE, Levine DM, Salmon JE (2006). Preclinical carotid atherosclerosis in patients with rheumatoid arthritis. Ann Intern Med.

